# Squamous Cell Carcinoma of the Lung with Metastasis to the GI Tract Associated with EGFR Exon 19 Deletion

**DOI:** 10.1155/2013/874836

**Published:** 2013-09-19

**Authors:** Glen J. Weiss, Agnes K. Liman, Jeffrey Allen, Philip Y. Cheung, Rajesh N. Kukunoor

**Affiliations:** ^1^Cancer Treatment Centers of America, Goodyear, AZ 85338, USA; ^2^The Translational Genomics Research Institute, Phoenix, AZ 85004, USA; ^3^CRAB Clinical Trials Consortium, Seattle, WA 98101, USA; ^4^Veterans Affairs Pittsburgh Healthcare System, Pittsburgh, PA 15206, USA; ^5^University of Pittsburgh School of Medicine, Pittsburgh, PA 15261, USA; ^6^Humboldt Medical Specialists, Eureka, CA 95501, USA; ^7^Ironwood Cancer and Research Centers, Phoenix, AZ 85028, USA

## Abstract

We describe three confirmed cases of squamous cell carcinoma (SCC) of the lung with metastasis to the gastrointestinal (GI) tract, with two having epidermal growth factor receptor (EGFR) exon 19 deletions in all available specimens. One of these patients received EGFR tyrosine kinase directed therapy for a brief period with some symptom relief. Consideration of EGFR exon 19 mutation testing in SCC of the lung, particularly for those with GI tract metastasis, may identify this potentially drug-targetable entity.

## 1. Introduction

Squamous cell carcinoma (SCC) of the lung metastasizing to the GI tract is an uncommon occurrence [1–4]. Epidermal growth factor receptor (EGFR) mutations have been identified in approximately 9% of metastatic SCC of the lung in one series [5]. We report on three patients with metastatic SCC of the lung metastasizing to the gastrointestinal (GI) tract, two of whom had tumor with a confirmed EGFR exon 19 deletion.

## 2. Case Presentations

**Case 1**

A 39-year-old never-smoker man presented with cough, headaches, night sweats, 7-pound weight loss, and constipation for several weeks and was found to have multifocal metastases involving the lung, brain, and colon. A diagnostic colonoscopy was performed, and pathology confirmed metastatic SCC. Additional samples from the lung obtained by bronchoscopy demonstrated SCC consistent with a primary nonsmall cell lung cancer (NSCLC). He then underwent craniotomy and resection for a solitary cerebellar metastasis with pathology consistent with metastatic SCC of the lung. Subsequently, he was treated with cisplatin and gemcitabine, followed by vinorelbine and docetaxel for up to 3 cycles before developing extracranial disease progression. Tissues from the lung, brain, and colon underwent independent expert pathology review and confirmed metastatic SCC of the lung. Because of his never-smoker status, the brain metastasis sample was sent for EGFR mutation testing (exons 18–21) and found to have an EGFR exon 19 deletion. The patient was started on erlotinib and had a transient clinical response with resolution of night sweats and 4-pound weight gain. Three months later, progression by radiographic evidence of bone metastases was observed, and he passed away three weeks later. No postprogression sample was available for analysis.

**Case 2**

A 79-year-old man with a 20-pack-year smoking history who was diagnosed with a 6.2 cm stage IIA T2bN0M0 SCC lung cancer underwent surgical resection. He also had a remote history of transitional cell carcinoma of the bladder. Adjuvant chemotherapy was not offered. Nearly 10 months later a colon metastasis was detected, and biopsy of the tumor was consistent with SCC of the lung. He subsequently developed brain metastasis and died approximately 28 months after his NSCLC diagnosis. Both the lung primary and colon metastasis were tested for and confirmed the presence of an EGFR exon 19 deletion.

**Case 3**

A 60-year-old man with an 80-pack-year smoking history and a periampullary cancer status after Whipple resection approximately 17 years earlier presented with decreased caliber of his bowel movements. A colonoscopy revealed a nearly obstructing distal colonic mass. While undergoing further workup, a lung mass was identified and biopsied confirming SCC of the lung. Immunohistochemical (IHC) staining was positive for CK5/6 and p63 and negative for CA19-9 and TTF-1. A laparoscopic segmental resection confirmed metastatic SCC of the lung. IHC staining of this specimen was positive for CK5/6, CK7, and p63 and negative for CK20 and CDX-2. Unfortunately, the patient had a prolonged hospital course with respiratory complications and development of abscesses and died approximately 6 weeks later. Available tumor from the sigmoid colon was tested for an EGFR exon 19 deletion, and an exon 19 deletion was not identified.

Table 1 outlines the available tumor samples from all three cases that were assessed for EGFR exon 19 deletion. 

## 3. Discussion

Of the three confirmed SCC of the lung with metastasis to the GI tract, two had EGFR exon 19 deletions. We did not detect any discordance for the mutation findings, as both primary and metastatic tumor from the first two cases had the EGFR exon 19 deletions. This suggests that EGFR exon 19 deletions are present in the initial primary tumor clone that has metastatic potential. While Case 3 did not have a detected EGFR exon 19 deletion, it is unlikely that if we had available primary tumor tissue, this deletion would be detected. Because of limitations of tumor tissue sample for Cases 2 and 3, evaluation of EGFR mutation status was focused on exon 19 deletion.

One of two patients (Case 1) received EGFR tyrosine kinase directed therapy for a brief period with some symptom relief. One can only speculate that if the EGFR exon 19 mutation was identified earlier in his disease course, there may have been improved clinical benefit.

## 4. Conclusion

Consideration of EGFR exon 19 mutation testing in SCC of the lung, particularly for those with GI tract metastasis, may identify this potentially drug-targetable entity.

## Figures and Tables

**Table 1:** Analysis of tumor samples.

	Location of tumor sample	Presence of EGFR exon 19 deletion (Y/N)
Case 1	Lung, cerebellar, mediastinal lymph nodes from stations 2, 7, 10R, and 4R, and colon	Y in all 7 samples
Case 2	Lung and colon	Y in both samples
Case 3	Sigmoid colon	N

## References

[1] Carroll D, Rajesh PB (2001). Colonic metastases from primary squamous cell carcinoma of the lung. *European Journal of Cardiothoracic Surgery*.

[2] Yang C-J, Hwang J-J, Kang W-Y (2006). Gastro-intestinal metastasis of primary lung carcinoma: clinical presentations and outcome. *Lung Cancer*.

[3] Stinchcombe TE, Socinski MA, Gangarosa LM, Khandani AH (2006). Lung cancer presenting with a solitary colon metastasis detected on positron emission tomography scan. *Journal of Clinical Oncology*.

[4] Kim SY, Ha HK, Park SW (2009). Gastrointestinal metastasis from primary lung cancer: CT findings and clinicopathologic features. *American Journal of Roentgenology*.

[5] Cadranel J, Mauguen A, Faller M (2012). Impact of systematic EGFR and KRAS mutation evaluation on progression-free survival and overall survival in patients with advanced non-small-cell lung cancer treated by erlotinib in a French prospective cohort (ERMETIC project—part 2). *Journal of Thoracic Oncology*.

